# Di-n-butyltin(IV) Complexes Derived from Heterocyclic β-diketones and N-Phthaloyl Amino Acids: Preparation, Biological Evaluation, Structural Elucidation Based upon Spectral [IR, NMR (^1^H, ^13^C, ^19^F and ^119^Sn)] Studies

**DOI:** 10.1155/BCA.2005.201

**Published:** 2005

**Authors:** Anurag Joshi, Shashi Verma, R. B. Gaurb, R. R. Sharma

**Affiliations:** 1 Department of Chemistry University of Rajasthan Jaipur 302 004 India; 2 Department of Plant Pathology Agricultural Research Station Rajasthan Agricultural University Sriganganagar 335 001 India

**Keywords:** Di-n-butyltin(IV) dichloride, heterocyclic β-diketones, N-phthaloyl amino acids, antimicrobial testing, spectral studies, micro organisms

## Abstract

Stable, six coordinated Bu_2_SnLA type complexes have been prepared [where LH = RCOC:C(OH)N(C_6_ H_5_)N:CCH_3_; R = -4-F-C_6_H_4_-(L^1^H), R = -4-Cl-C_6_H_4_-(L^2^H), R= -4-Br-C_6_H_4_-(L^3^H), R=-CF_3_(L^4^H) and AH = C(O)C_6_ H_4_ C(O)NCHR'COOH; R'= -H(A^1^H), -CH_3_(A^2^H), -CH(CH_3_)_2_(A^3^H)] by the interaction of 1:1:1 molar ratios of di-n-butyltin(IV) dichloride with corresponding organic moieties in refluxing benzene using two moles of Et_3_N as a base. In these complexes LH and AH behave as bidentate and coordination is taking place through oxygen, this is inferred from IR and ^13^C NMR studies. These complexes possess tin atoms in skew trapezoidal bipyramidal geometry with the C-Sn-C angles ranging from 149.88^°^ to 156.84^°^. Some of these complexes with their corresponding organic moieties (LH, AH) were tested for their antimicrobial activities.


### Di-n-butyltin(IV) Complexes Derived from Heterocyclic β-diketones and N-Phthaloyl Amino Acids : Preparation, Biological Evaluation, Structural Elucidation Based upon Spectral [IR, NMR ($^{1}H$, $^{13}C$, $^{19}F$ and $^{119}Sn$)] Studies.

Anurag Joshi[a,*], Shashi Verma[a], R.B. Gaur[b], R.R. Sharma[b]

[a]*Department of Chemistry, University of Rajasthan, Jaipur-302 004, India*
[b]*Department of Plant Pathology, Agricultural Research Station, Rajasthan Agricultural University, Sriganganagar - 335 001, India*

### ABSTRACT

Stable, six coordinated $Bu_2SnLA$ type complexes have been prepared [where LH = RCOC:C(OH)N($C_6H_5$)N:CCH$_3$; R = -4-F-$C_6H_4$-(L[1]H), R = -4-Cl-$C_6H_4$-(L[2]H), R= -4-Br-$C_6H_4$-(L[3]H), R= -$CF_3$(L[4]H) and AH = C(O)$C_6H_4$C(O)NCHR'COOH; R'= -H(A[1]H), -$CH_3$(A[2]H), -CH($CH_3$)$_2$(A[3]H)] by the interaction of 1:1:1 molar ratios of di-n-butyltin(IV) dichloride with corresponding organic moieties in refluxing benzene using two moles of $Et_3N$ as a base. In these complexes LH and AH behave as bidentate and coordination is taking place through oxygen, this is inferred from IR and $^{13}C$ NMR studies. These complexes possess tin atoms in skew trapezoidal bipyramidal geometry with the C-Sn-C angles ranging from 149.88° to 156.84°. Some of these complexes with their corresponding organic moieties (LH ,AH) were tested for their antimicrobial activities.

**Keywords:** Di-n-butyltin(IV) dichloride, heterocyclic β-diketones, N-phthaloyl amino acids, antimicrobial testing, spectral studies, micro organisms.

### INTRODUCTION

A large number of organotin(IV) complexes using a variety of organic ligands /1-6/ have been synthesised and characterised in recent years due to their significant biological activities /7-9/. Some of these derivatives have been tested for *in vitro* activity on different type of tumour cells /10-12/. They also possess significant applications in different fields such as lubricating agents /13/, boat paint additives to prevent

* Corresponding author
E-mail: saket_apsilon@rediffmail.com; Fax : +91-141-2704834

attack by micro organisms/14/, catalysts /15,16/, polymers /17/ and organic synthesis /18/. In continuation of our work on mixed ligand dibutyltin(IV) and dicyclopentadienyltitanium(IV) complexes derived from heterocyclic β-diketones and N-phthaloyl amino acids /19,20/, now an attempt has been made to synthesise mixed ligand dibutyltin(IV) complexes using different types of substituents on these organic moieties.

### MATERIALS AND METHODS

All experimental works were performed under a moisture free atmosphere. Chemicals and solvents used were dried and purified by standard methods /21/. The substituted heterocyclic β-diketones and N-phthaloyl amino acids have been synthesised by the literature procedures /22,23/.

### Synthesis of $Bu_2Sn[FC_6H_4COC{:}C(O)N(C_6H_5)N{:}CCH_3][O_2CCH_2NC(O)C_6H_4C(O)]$

Benzene solution (30 ml) of ($L^1H$), 1-Phenyl-3-methyl-4-(4′-Fluorobenzoyl)-5-Pyrazolone (1.00 g, 3.4 mmol); ($A^1H$), 1,3-dihydro-1,3-dioxo-2H-isoindole-2-acetic acid (0.69 g, 3.4 mmol) and $Et_3N$ (0.68 g, 6.8 mmol) were added dropwise to a benzene solution (30 ml) of di-n-butyltin(IV) dichloride (1.03 g, 3.4 mmol). After refluxing this solution ~8-10 h., the precipitated $Et_3N.HCl$ was filtered off and the volatile components of the filtrate were removed under reduced pressure to yield a coloured solid. All other derivatives were synthesised by the same procedure and the preparative and analytical data are summarised in Table 1.

### Analytical Methods and Spectral Measurements

IR Spectra were recorded (4000-200 $cm^{-1}$) as KBr pellets on Nicolet Magna 550 spectrophotometer. Microanalyses (C,H,N) were performed using a Perkin Elmer 2400 CHNS/O analyzer. $^1H$, $^{19}F$ and $^{13}C$ NMR spectra were recorded in $CDCl_3$ and $CHCl_3$ solutions, respectively, on a JEOL FX-90 Q FT spectrometer. $^{119}Sn$ NMR spectra were recorded in $CHCl_3$ solution. Melting points were determined in sealed capillaries. Molecular weights were determined cryoscopically in benzene.

### Antimicrobial Screening

#### *Qualitative Antimicrobial Assay*

The antimicrobial activities of LH,AH and their metal complexes were tested against six pathogenic micro organisms (i) *Staphylococcus aureus* (Gram positive bacteria), (ii) *Streptococcus viridans* (Gram positive bacteria), (iii) *Escherichia coli* (Gram Negative bacteria), (iv) *Fusarium oxysporium* (Fungus), (v) *Alternaria alternata* (Fungus), (vi) *Alternaria solani* (Fungus). The culture was maintained by the reported procedure /24/. The antimicrobial activity of the extracts was qualitatively determined by a modified disc diffusion method /25/. A lawn of micro-organisms was prepared by pipetting and evenly spreading inoculum ($10^5$-$10^6$ c.f.u./$cm^3$) [c.f.u. = colony forming units] onto agar set in Petri dishes, using nutrient agar (NA) for

**Table 1**

Synthetic and analytical data of di-n-butyltin(IV) complexes.

| S. No. | Reagents in g (mmol) | | | | Product Formula | MP (°C) | % Elemental Analysis Found (Calcd.) | | | | Molecular Wt. Found (Calcd.) |
|---|---|---|---|---|---|---|---|---|---|---|---|
| | $Bu_2SnCl_2$ | LH | AH | $Et_3N$ | | | C | H | N | Sn | |
| **1.** | 1.03 (3.4) | $L^1H$ 1.00 (3.4) | $A^1H$ 0.69 (3.4) | 0.68 (6.8) | $C_{35}H_{36}N_3O_6FSn$ $Bu_2SnL^1A^1$ | 110 | 57.31 (57.40) | 4.65 (4.95) | 5.60 (5.74) | 16.19 (16.21) | 729 (732.39) |
| **2.** | 0.95 (3.1) | $L^1H$ 0.92 (3.1) | $A^2H$ 0.68 (3.1) | 0.63 (6.2) | $C_{36}H_{38}N_3O_6FSn$ $Bu_2SnL^1A^2$ | 135 | 57.80 (57.93) | 5.12 (5.13) | 5.61 (5.63) | 15.87 (15.90) | 742 (746.41) |
| **3.** | 0.85 (2.8) | $L^1H$ 0.82 (2.8) | $A^3H$ 0.69 (2.8) | 0.56 (5.5) | $C_{38}H_{42}N_3O_6FSn$ $Bu_2SnL^1A^3$ | 117 | 58.90 (58.93) | 5.40 (5.47) | 5.41 (5.43) | 15.30 (15.33) | 770 (774.46) |
| **4.** | 0.89 (2.9) | $L^2H$ 0.91 (2.9) | $A^1H$ 0.62 (2.9) | 0.59 (5.8) | $C_{35}H_{36}N_3O_6ClSn$ $Bu_2SnL^2A^1$ | 149 | 56.00 (56.14) | 4.80 (4.85) | 5.59 (5.61) | 15.78 (15.85) | 740 (748.84) |
| **5.** | 1.42 (4.7) | $L^2H$ 1.46 (4.7) | $A^2H$ 1.02 (4.7) | 0.94 (9.3) | $C_{36}H_{38}N_3O_6ClSn$ $Bu_2SnL^2A^2$ | 125 | 56.60 (56.68) | 5.01 (5.02) | 5.50 (5.51) | 15.49 (15.56) | 758 (762.87) |
| **6.** | 0.74 (2.4) | $L^2H$ 0.76 (2.4) | $A^3H$ 0.60 (2.4) | 0.49 (4.8) | $C_{38}H_{42}N_3O_6ClSn$ $Bu_2SnL^2A^3$ | 131 | 57.70 (57.71) | 5.34 (5.35) | 5.29 (5.31) | 15.00 (15.01) | 788 (790.92) |
| **7.** | 1.13 (3.7) | $L^3H$ 1.32 (3.7) | $A^1H$ 0.76 (3.7) | 0.75 (7.4) | $C_{35}H_{36}N_3O_6BrSn$ $Bu_2SnL^3A^1$ | 140 | 52.89 (52.99) | 4.50 (4.57) | 5.29 (5.30) | 14.81 (14.96) | 785 (793.29) |
| **8.** | 0.83 (2.7) | $L^3H$ 0.97 (2.7) | $A^2H$ 0.59 (2.7) | 0.55 (5.4) | $C_{36}H_{38}N_3O_6BrSn$ $Bu_2SnL^3A^2$ | 107 | 53.48 (53.56) | 4.68 (4.74) | 5.19 (5.20) | 14.63 (14.70) | 798 (807.32) |
| **9.** | 1.07 (3.5) | $L^3H$ 1.25 (3.5) | $A^3H$ 0.87 (3.5) | 0.71 (7.0) | $C_{38}H_{42}N_3O_6BrSn$ $Bu_2SnL^3A^3$ | 145 | 54.60 (54.64) | 5.03 (5.07) | 5.00 (5.03) | 14.18 (14.21) | 830 (835.37) |
| **10.** | 0.84 (2.7) | $L^4H$ 0.74 (2.7) | $A^1H$ 0.56 (2.7) | 0.55 (5.4) | $C_{30}H_{32}N_3O_6F_3Sn$ $Bu_2SnL^4A^1$ | 135 | 50.07 (51.02) | 4.50 (4.57) | 5.90 (5.95) | 16.67 (16.81) | 694 (706.30) |
| **11.** | 0.92 (3.0) | $L^4H$ 0.82 (3.0) | $A^2H$ 0.66 (3.0) | 0.61 (6.0) | $C_{31}H_{34}N_3O_6F_3Sn$ $Bu_2SnL^4A^2$ | 163 | 51.58 (51.69) | 4.70 (4.76) | 5.81 (5.83) | 16.45 (16.48) | 710 (720.32) |
| **12.** | 0.71 (2.3) | $L^4H$ 0.63 (2.3) | $A^3H$ 0.57 (2.3) | 0.47 (4.6) | $C_{33}H_{38}N_3O_6F_3Sn$ $Bu_2SnL^4A^3$ | 142 | 52.87 (52.96) | 5.10 (5.12) | 5.59 (5.61) | 15.80 (15.86) | 740 (748.38) |

bacteria. Whatman No. 1 filter paper discs of 6 mm diameter were impregnated with the stock solutions of the complexes (100 mg $cm^{-3}$) in DMSO and dried under sterile condition. The dried discs were then placed on the previously inoculated agar surface. The plates were inverted and incubated for 24 h. at 30°C. Antimicrobial activity was indicated by the presence of clear inhibition zones around the discs.

### *Quantitative Antimicrobial Assay*

Substances showing positive antimicrobial activity via the disc diffusion assay were subjected to the broth dilution method for the quantitative measurement of microbiostatic (inhibitory) activity as described by Hufford and Clark /26/. The lowest concentration that completely inhibited visible microbial growth was recorded as the minimum inhibitory concentration (MIC, μg $cm^{-3}$) of the species in DMSO. Both Nystatin and Kanamycin were used as positive controls.

### RESULTS AND DISCUSSION

The reactions of $Bu_2SnCl_2$ with heterocyclic β-diketones (LH) (**Fig. 1a**), N-phthaloyl amino acids (AH) (**Fig. 1b**) and a base $Et_3N$ in 1:1:1:2 molar ratios in refluxing benzene solution afforded mixed ligand di-n-butyltin(IV) complexes.

$Bu_2SnCl_2$+RCOC:C(OH)N($C_6H_5$)N:$CCH_3$ + C(O)$C_6H_4$C(O)NCHR'COOH +2$Et_3$N

$C_6H_6$, Reflux → $Bu_2Sn$[RCOC:C(O)N($C_6H_5$)N:$CCH_3$] [$O_2$CCHR'NC(O)$C_6H_4$C(O)]

+2$Et_3$N.HCl↓

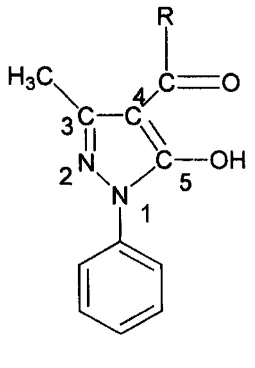

R = -4-F-$C_6H_4$-($L^1H$)

R = -4-Cl-$C_6H_4$-($L^2H$)

R = -4-Br-$C_6H_4$-($L^3H$)

R = -$CF_3$($L^4H$);

**Fig. 1a:** Structure of heterocyclic β-diketones (LH)

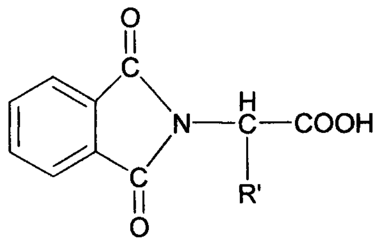

R' = -H($A^1H$)

R' = -$CH_3$($A^2H$)

R' = -CH($CH_3$)$_2$($A^3H$)

**Fig. 1b:** Structure of N-phthaloyl amino acids (AH)

| | | | |
|---|---|---|---|
| R = -4-F-$C_6H_4$-, | R′= -H, | $Bu_2SnL^1A^1$ | Complex **1** |
| R = -4-F-$C_6H_4$-, | R′= -$CH_3$, | $Bu_2SnL^1A^2$ | Complex **2** |
| R = -4-F-$C_6H_4$-, | R′= - $CH(CH_3)_2$, | $Bu_2SnL^1A^3$ | Complex **3** |
| R = -4-Cl-$C_6H_4$-, | R′= -H, | $Bu_2SnL^2A^1$ | Complex **4** |
| R = -4-Cl-$C_6H_4$-, | R′= -$CH_3$, | $Bu_2SnL^2A^2$ | Complex **5** |
| R = -4-Cl-$C_6H_4$-, | R′= - $CH(CH_3)_2$, | $Bu_2SnL^2A^3$ | Complex **6** |
| R = -4-Br-$C_6H_4$-, | R′= -H, | $Bu_2SnL^3A^1$ | Complex **7** |
| R = -4-Br-$C_6H_4$-, | R′= -$CH_3$, | $Bu_2SnL^3A^2$ | Complex **8** |
| R = -4-Br-$C_6H_4$-, | R′= - $CH(CH_3)_2$, | $Bu_2SnL^3A^3$ | Complex **9** |
| R = -$CF_3$, | R′= -H, | $Bu_2SnL^4A^1$ | Complex **10** |
| R = -$CF_3$, | R′= -$CH_3$, | $Bu_2SnL^4A^2$ | Complex **11** |
| R = -$CF_3$, | R′= -$CH(CH_3)_2$, | $Bu_2SnL^4A^3$ | Complex **12** |

After filtering off the $Et_3N.HCl$ and stripping off the volatile fraction from filtrate, all these derivatives were obtained as coloured solids. These derivatives were found to be soluble in common organic solvents and were recrystallised from $CHCl_3$/Pet.-ether mixture. The plausible structures of these newly synthesised mixed ligand complexes of di-n-butyltin(IV) have been proposed on the basis of spectral studies.

### IR Spectra

A comparison of the IR spectra of these mixed ligand di-n-butyltin(IV) complexes has been found to be helpful in providing structural assignments of the complexes. νOH absorption has been found to be absent in the region 3400 – 2600 $cm^{-1}$, showing that both the organic moieties are bonded through deprotonated forms which is supported by the presence of νSn-O vibrations in the region 650 $\pm$ 60 $cm^{-1}$ /27,28/. A band appearing at ~1760 $cm^{-1}$ due to $\nu CO_{(asym)}$ vibration in the spectra of N-phthaloyl amino acids (AH) does not show any significant shift, suggesting the non-involvement of this group (imido CO) in bonding. A broad band centered at ~1702 $cm^{-1}$ in the IR spectra of AH may be assigned to [$\nu CO_{(sym)}$ + $\nu COO_{(asym)}$] vibrations splits into two after complex formation . The sharp band at ~ 1702 $cm^{-1}$ and a medium intensity band at 1640 – 1610 $cm^{-1}$ may be assigned to $\nu CO_{(sym)}$ and $\nu COO_{(asym)}$ vibrations, respectively. The splitting of this band into two indicates the bidentate behaviour of AH/29,30/. The values of $\Delta\nu[\nu COO_{(asym)}-\nu COO_{(sym)}]$ can be calculated by the reported procedure/19/ and have been obtained in the range 215 – 230 $cm^{-1}$, further suggesting the chelating nature of carboxylate group of AH/31/.

νC=O stretching vibrations of heterocyclic β-diketones (LH) appear as a strong band in the region 1660 – 1600 $cm^{-1}$; this band is shifted towards lower frequency of the order of ~50-15 $cm^{-1}$ and may be assigned to νC ⋯⋯ O vibration. This shifting of νCO band in β-diketone moiety suggests the coordination through carbonyl oxygen and its bidentate nature /27/. No significant shift in the positions of bands at ~1570 and 1590 $cm^{-1}$ has been observed. These bands may be assigned to ν(C=C / C =N) and phenyl vibrations, respectively. A medium intensity band in the region 610 – 590 $cm^{-1}$ has been assigned to Sn-C vibration /32/.

The presence of only one Sn-C band indicates that the two butyl groups are in trans axial position /33/.

According to group theoretical predictions the trans- $SnO_4C_2$ system should exhibit one Sn-O and one Sn-C vibration and the cis isomer two Sn-C and four Sn-O stretching vibrations in the IR spectra /34/ (**Fig. 2**). The appearance of one Sn-C and one Sn-O stretching vibration in the IR spectra of these mixed ligand di-n-butyltin(IV) complexes suggests that two butyl groups are in trans configuration.

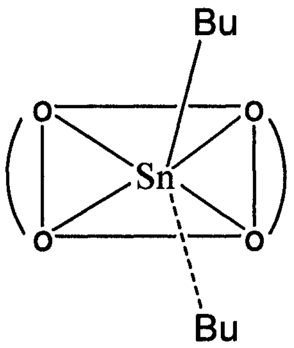

**Fig. 2**

### $^1$H NMR Spectra

The $^1$H NMR spectra (Table 2) of these di-n-butyltin(IV) mixed ligand complexes show interaction of both organic moieties towards central tin atom through deprotonated form as inferred from disappearance of -OH signals. Aromatic protons appear as a multiplet in the region δ 7.02 – 8.46 ppm. The signals due to butyl protons attached to tin exhibit a complex pattern in the region δ 2.63 - 0.33 ppm for **2, 3, 5, 6, 8, 9, 11** and **12**. The $^2J(^{119}Sn-^1H)$ coupling constant values in these derivatives could not be observed as signals due to methyl groups of N-phthaloyl amino acids (except $A^1H$) are merged with the signals due to butyl groups attached to the central tin atom. In the derivatives derived from LH and $A^1H$ (**1,4,7**), protons of butyl groups appear as triplet at δ 0.75 – 0.89 ppm and two multiplets at δ 1.28 – 1.66 ppm, δ 1.23 – 1.98 ppm due to $CH_3$ and $CH_2$ groups, respectively. The $^2J$ ($^{119}Sn-^1H$).values of 93, 96 and 97 Hz observed for **1,4** and **7** are found to be very close to the reported value (~ 101 Hz) for six coordinated diorganotin(IV) complexes /35,36/.

According to the literature, the use of the Lockhart-Manders equation /37/ shown below

$$\theta = 0.0161\{^2J(^{119}Sn-^1H)\}^2 - 1.32\{^2J(^{119}Sn-^1H)\} + 133.4$$

yields C-Sn-C angles of 149.88°, 155.05° and 156.84° for **1,4** and **7**, respectively, indicating the presence of two butyl groups attached with tin atom in trans position with distorted octahedral (skew trapezoidal bipyramidal) geometry /38/. Other $^1$H NMR spectral data are summarised in Table 2.

### $^{13}$C NMR Spectra

$^{13}$C NMR spectra of di-n-butyltin(IV) complexes have been recorded in $CHCl_3$ solution and are tabulated as Table 3. A small shift has been noticed in the positions of $C_5$ and COO carbons of LH and AH,

**Table 2**

[1]H NMR spectral data of LH,AH and their corresponding di-n-butyltin(IV) complexes (in δ ppm)[a].

| Compound No. | Ligands and Complexes | RCOC:C(OH)N($C_6H_5$)N:CCH$_3$ (LH) | | | | COC$_6$H$_4$CONCHR'COOH (AH) | | | | | Sn-Bu |
|---|---|---|---|---|---|---|---|---|---|---|---|
| | | OH | Ring Methyl | -N-$C_6H_5$ | R | OH | -$C_6H_4$- | CH | $CH_2$ | $CH_3$ | |
| | $L^1H$ | 9.00 br (s) | 2.15(s) | 7.22-7.88(m) | * | | | | | | |
| | $A^1H$ | | | | | ** | 8.05-7.60(m) | - | 4.47(s) | - | |
| 1. | $Bu_2SnL^1A^1$ | - | 2.17(s) | 7.10-8.05(m) | * | - | * | - | 4.40(s) | - | 0.81(t)(7.6Hz) 1.28(m) 1.5-1.75(m) |
| | $A^2H$ | | | | | 9.27(s) | 7.87(m) | 5.08(q) | - | 1.74(d) | |
| 2. | $Bu_2SnL^1A^2$ | - | 2.16(s) | 7.16-8.10(m) | * | - | * | 4.7(q) | - | *** | 2.47-0.33(m) |
| | $A^3H$ | | | | | 8.94(s) | 7.80(m) | 4.60(d) 2.70(st) | - | 1.15(d) 0.95(d) | |
| 3. | $Bu_2SnL^1A^3$ | - | 2.15(s) | 7.08-8.43(m) | * | - | * | 4.59(d) 2.70(st) | - | *** | 2.38-0.54(m) |
| | $L^2H$ | 8.70 br (s) | 2.13(s) | 7.32-7.88(m) | * | | | | | | |
| 4. | $Bu_2SnL^2A^1$ | - | 2.10(s) | 7.03-8.24(m) | * | - | * | - | 4.39(s) | - | 0.75(t)(7.3Hz) 1.31(m) 1.5-1.98(m) |
| 5. | $Bu_2SnL^2A^2$ | - | 2.04(s) | 7.10-8.23(m) | * | - | * | 5.00(q) | - | *** | 2.63-0.37(m) |
| 6. | $Bu_2SnL^2A^3$ | - | 1.98(s) | 7.03-8.19(m) | * | - | * | 4.56(d) 2.69(st) | - | *** | 2.54-0.66(m) |
| | $L^3H$ | 11.5 br (s) | 2.12(s) | 7.30-7.86(m) | * | | | | | | |
| 7. | $Bu_2SnL^3A^1$ | - | 2.07(s) | 7.02-8.16(m) | * | - | * | - | 4.41(s) | - | 0.89(t)(7.2Hz) 1.66(m) 1.23-1.38(m) |
| 8. | $Bu_2SnL^3A^2$ | - | 1.94(s) | 7.10-8.33(m) | * | - | * | 5.06(q) | - | *** | 2.50-0.63(m) |
| 9. | $Bu_2SnL^3A^3$ | - | 1.90(s) | 7.17-8.46(m) | * | - | * | 4.54(d) 2.69(st) | - | *** | 2.53-0.33(m) |
| | $L^4H$ | 7.13(s) | 2.47(s) | 7.35-.78(m) | - | | | | | | |
| 10. | $Bu_2SnL^4A^1$ | - | 2.33(s) | 7.12-.07(m) | - | - | * | - | 4.43(s) | - | 0.75(m), 1.28(m), 1.60(m) |
| 11. | $Bu_2SnL^4A^2$ | - | 2.28(s) | 7.10-.00(m) | - | - | * | 4.7(q) | - | *** | 2.58-0.36 (m) |
| 12. | $Bu_2SnL^4A^3$ | - | 2.37(s) | 7.03-.24(m) | - | - | * | 4.57(d) 2.68(st) | - | *** | 2.09-0.54 (m) |

*merged with -N-$C_6H_5$ region

**$NH^+$ signal appeared at δ 4.2 ppm

***merged with butyl region

[a]s : singlet; d : doublet; q : quartet; st : septet; m : multiplet

**Table 3**

$^{13}C$ NMR spectral data of LH,AH with their di-n-butyltin(IV) complexes (in δ ppm).

| Compound No. | Ligands and Complexes | RCOC:C(OH)N($C_6H_5$)N:CCH$_3$ (LH) | | | | | | | COC$_6H_4$CONCHR'COOH (AH) | | | | | | Sn-Butyl |
|---|---|---|---|---|---|---|---|---|---|---|---|---|---|---|---|
| | | $CH_3$ | C=O | $C_5$ | $C_4$ | $C_3$ | -N-$C_6H_5$ | R | COO | CO | CH | $CH_2$ | $CH_3$ |  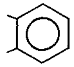 | |
| | $L^1H$ | 15.9 | 190.9 | 162.1 | 103.4 | 147.6 | 151.7, 137.4, 131.4, 129.7, 129.3, 126.5, 121.7, 114.8 | * | | | | | | | |
| | $A^1H$ | | | | | | | | 170.46 | 167.29 | - | 40.25 | - | 136.03, 134.29, 125.25 | |
| 1. | $Bu_2SnL^1A^1$ | 16.0 | 191.3 | 163.3 | 104.7 | 147.8 | 151.9, 138.4, 132.2, 130.9, 130.3, 126.9, 121.9, 115.8 | * | 175.86 | 167.47 | - | 39.38 | - | 133.87, 132.91, 125.78 | 13.42, 26.63, 27.49, 28.54 |
| | $A^2H$ | | | | | | | | 174.87 | 167.28 | 47.13 | - | 14.85 | 133.14, 131.59, 123.41 | |
| 2. | $Bu_2SnL^1A^2$ | 16.3 | 191.7 | 163.7 | 104.2 | 147.8 | 152.4, 138.9, 132.7, 131.9, 131.6, 127.2, 122.4, 115.3 | * | 178.07 | 167.31 | 47.88 | - | 15.35 | 133.72, 132.49, 125.40 | 13.61, 26.29, 26.72, 28.44 |
| | $A^3H$ | | | | | | | | 174.44 | 167.67 | 57.43 28.34 | - | 20.86 19.45 | 134.24, 131.81, 123.25 | |

**Table 3** (continued)

$^{13}C$ NMR spectral data of LH,AH with their di-n-butyltin(IV) complexes (in δ ppm).

| Compound No. | Ligands and Complexes | RCOC:C(OH)N($C_6H_5$)N:CCH$_3$ (LH) | | | | | | | $COC_6H_4CONCHR'COOH$ (AH) | | | | | | Sn-Butyl |
|---|---|---|---|---|---|---|---|---|---|---|---|---|---|---|---|
| | | $CH_3$ | C=O | $C_5$ | $C_4$ | $C_3$ | -N-$C_6H_5$ | R | COO | CO | CH | $CH_2$ | $CH_3$ |  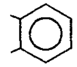 | |
| **3.** | $Bu_2SnL^1A^3$ | 16.1 | 191.0 | 163.2 | 104.1 | 147.9 | 151.9, 137.4, 131.8, 131.4, 129.9, 126.8, 121.9, 116.0 | * | 179.32 | 167.60 | 58.09 28.63 | - | 21.72 19.87 | 134.83, 132.41, 125.87 | 13.83, 26.39, 26.89, 27.40 |
| | $L^2H$ | 16.4 | 191.6 | 161.5 | 104.0 | 148.1 | 147.6, 138.0, 129.1, 129.0, 128.8, 126.7, 126.2, 120.8 | * | | | | | | | |
| **4.** | $Bu_2SnL^2A^1$ | 16.7 | 193.9 | 162.9 | 104.9 | 148.6 | 148.3, 138.2, 129.7, 129.4, 128.8, 127.1, 126.9, 120.9 | * | 175.46 | 167.20 | - | 39.42 | - | 133.54, 135.61, 125.45 | 13.88, 25.78, 27.69, 27.71 |
| **5.** | $Bu_2SnL^2A^2$ | 17.0 | 192.0 | 162.0 | 104.3 | 148.3 | 148.7, 138.4, 129.7, 129.2, 128.9, 127.3, 126.8, 121.2 | * | 178.87 | 167.29 | 47.63 | - | 15.31 | 133.14, 132.98, 123.49 | 13.58, 26.61, 26.90, 28.71 |
| **6.** | $Bu_2SnL^2A^3$ | 17.3 | 192.8 | 162.7 | 104.8 | 148.9 | 147.9, 138.1, 129.9, 129.5, 128.8, 127.5, 126.5, 121.4 | * | 180.34 | 167.64 | 58.69 28.32 | - | 21.32 19.75 | 134.32, 131.83, 123.25 | 13.57, 26.41, 26.63, 28.41 |
| | $L^3H$ | 15.9 | 191.2 | 161.1 | 103.5 | 147.7 | 150.2, 137.9, 131.1, 129.5, 129.1, 126.6, 125.3, 118.4 | * | | | | | | | |

**Table 3** (continued)

$^{13}C$ NMR spectral data of LH,AH with their di-n-butyltin(IV) complexes (in δ ppm).

| Compound No. | Ligands and Complexes | RCOC:C(OH)N($C_6H_5$)N:CCH$_3$ (LH) | | | | | | | COC$_6$H$_4$CONCHR'COOH (AH) | | | | | | Sn-Butyl |
|---|---|---|---|---|---|---|---|---|---|---|---|---|---|---|---|
| | | $CH_3$ | C=O | $C_5$ | $C_4$ | $C_3$ | -N-$C_6H_5$ | R | COO | CO | CH | $CH_2$ | $CH_3$ |  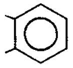 | |
| 7. | $Bu_2SnL^3A^1$ | 16.1 | 192.4 | 163.0 | 104.7 | 147.9 | 151.4, 138.0, 131.7, 130.6, 130.1, 127.8, 126.8, 119.5 | * | 176.73 | 167.40 | - | 39.07 | - | 133.54, 132.18, 125.61 | 13.09, 26.52, 26.81, 28.63 |
| 8. | $Bu_2SnL^3A^2$ | 16.7 | 193.1 | 162.7 | 104.9 | 147.6 | 151.7, 138.4, 131.9, 130.4, 129.8, 127.0, 126.0, 119.9 | * | 179.62 | 167.42 | 47.91 | - | 15.47 | 133.62, 132.41, 124.89 | 13.81, 26.72, 27.18, 28.54 |
| 9. | $Bu_2SnL^3A^3$ | 16.5 | 192.9 | 162.9 | 105.2 | 147.8 | 151.3, 138.7, 131.4, 130.8, 129.9, 126.9, 126.5, 119.3 | * | 180.04 | 167.54 | 58.32, 28.61 | - | 21.39, 19.53 | 134.81, 131.72, 125.50 | 13.70, 26.37, 27.21, 27.51 |
| | $L^4H$ | 14.8 (s) | 177.3 | 161.0 | 99.6 | 147.9 | 136.3, 129.3, 128.0, 121.4 | 116.2(q) | | | | | | | |
| 10. | $Bu_2SnL^4A^1$ | 15.6 (s) | 178.6 | 162.7 | 100.3 | 148.9 | 137.4, 130.0, 128.9, 122.3 | 116.8(q) | 176.32 | 167.34 | - | 38.72 | - | 136.09, 134.78, 125.63 | 13.47, 26.63, 26.78, 28.61 |
| 11. | $Bu_2SnL^4A^2$ | 15.0 (s) | 180.7 | 162.9 | 100.8 | 148.3 | 136.8, 129.9, 128.7, 121.9 | 117.4(q) | 177.97 | 167.56 | 47.52 | - | 15.11 | 133.72, 131.88, 124.43 | 13.32, 26.61, 27.53, 28.03 |
| 12. | $Bu_2SnL^4A^3$ | 15.9 (s) | 177.8 | 161.4 | 100.2 | 148.0 | 137.3, 130.4, 129.3, 122.5 | 116.4(q) | 176.82 | 167.81 | 58.61, 28.69 | - | 21.34, 20.58 | 135.60, 132.25, 123.87 | 13.42, 26.81, 27.92, 28.03 |

*merged with -N-$C_6H_5$ region respectively. These results are in agreement with earlier reported values /24/ suggesting the bidentate nature of LH and AH moieties. Important information related to the structure of coordination polyhedra of di-n-butyltin(IV) complexes can be obtained from the values of the coupling constants $^{1}J(^{119}Sn-^{13}C)$, because they are directly linked to the size of C-Sn-C angle (θ). $^{1}J$ $(^{119}Sn-^{13}C)$ coupling constant value found for **1** is 780 Hz, which supports the six coordination number for the central tin atom /39/. On the basis of the equation given by Holocek and Lycka /40/, the C-Sn-C angle in **1** is estimated to *ca*. 152.75°.

$$^{1}J = 9.99\theta - 746$$

This indicates that the two butyl groups occupy the trans position in the octahedral geometry /41/. An identical value of θ has been reported by Kepert /42/ suggesting the skew trapezoidal bipyramidal geometry. The $^{1}J$ (Sn-C) coupling constant values could not be observed for other complexes due to the low intensity of Bu-Sn carbon resonances which resulted in a poor signal/noise ratio /43/.

### $^{19}$F NMR Spectra

$^{19}$F NMR Spectra of $L^4H$ and corresponding di-n-butyltin(IV) complexes (**10**,**11** and **12**) have been recorded relative to $CFCl_3$. $L^4H$ gives a single fluorine resonance peak at δ -75.2 ppm while **10**,**11** and **12** show absorption peaks at δ -70.6 ppm, δ -71.4 ppm and δ -72.6 ppm, respectively. Similar downfield shifting in fluorine resonance chemical shift has been reported in literature /44/, which suggests the non-involvement of the fluorine atom in bonding.

### $^{119}$Sn NMR Spectra

These mixed ligand diorganotin(IV) complexes have been subjected to $^{119}$Sn NMR studies and their results have been cited in Table 4. These complexes show $^{119}$Sn NMR signals in the range δ -270.81 to -390.57 ppm. $^{119}$Sn NMR chemical shift values have been found to be influenced by the coordination number of tin atom /41/. These $^{119}$Sn NMR data are consistent with the earlier reported values /37,41,45/ for hexa coordinated diorganotin(IV) complexes.

### Antimicrobial Results

Preliminary screening for antimicrobial activities of the stock solutions of metal complexes and corresponding organic moieties (LH,AH) were performed qualitatively using the disc diffusion assay. Antimicrobial activities were measured from the diameter of clear inhibition zones caused by compounds. Four compounds (**1**,**4**,**7** and **10**) were found to yield clear inhibition zones around the discs (Table 5).

### *Qualitative Antimicrobial Activity Results*

Almost all the compounds were found to be more active than the heterocyclic β-diketones (LH), N-phthaloyl amino acids (AH) against all the organisms used. It may therefore, be concluded that the

**Table 4**

$^{119}Sn$ NMR spectral data of some mixed ligand di-n-butyltin(IV) complexes.

| Complex No. | Complex formula | Chemical shift values (in δ ppm) |
|---|---|---|
| | $Bu_2SnCl_2$ | 126.3[a] |
| **1** | $Bu_2SnL^1A^1$ | - 270.81 |
| **2** | $Bu_2SnL^1A^2$ | - 295.54 |
| **3** | $Bu_2SnL^1A^3$ | - 310.34 |
| **4** | $Bu_2SnL^2A^1$ | - 285.61 |
| **5** | $Bu_2SnL^2A^2$ | - 362.67 |
| **6** | $Bu_2SnL^2A^3$ | - 345.60 |
| **7** | $Bu_2SnL^3A^1$ | -318.59 |
| **8** | $Bu_2SnL^3A^2$ | -310.86 |
| **9** | $Bu_2SnL^3A^3$ | -298.42 |
| **10** | $Bu_2SnL^4A^1$ | -248.72 |
| **11** | $Bu_2SnL^4A^2$ | -385.42 |
| **12** | $Bu_2SnL^4A^3$ | -390.57 |

[a] Ref. 49

**Table 5**

Qualitative antimicrobial assay results [Inhibition zone (mm)].

| Compound | S.a. | S.v. | E.c. | F.o. | A.a. | A.s. |
|---|---|---|---|---|---|---|
| $L^1H$ | 30 | 28 | 26 | a | - | 31 |
| $L^2H$ | 33 | 35 | 29 | - | 39 | 45 |
| $L^3H$ | 37 | a | - | a | 46 | a |
| $L^4H$ | - | - | a | a | a | a |
| $A^1H$ | 34 | 28 | 34 | 37 | a | 42 |
| $Bu_2SnL^1A^1$ **(1)** | 42 | 48 | 26 | 36 | 47 | 48 |
| $Bu_2SnL^2A^1$ **(4)** | 37 | 34 | 29 | 41 | 32 | 38 |
| $Bu_2SnL^3A^1$ (7) | 33 | 42 | 32 | 48 | 34 | 39 |
| $Bu_2SnL^4A^1$ **(10)** | 21 | 22 | 18 | 28 | 32 | 32 |
| Kanamycin | 21 | 23 | 24 | | | |
| Nystatin | | | | 23 | 27 | 24 |

a = very weak active

S.a. = *Staphylococcus aureus* (gram positive bacteria); S.v. = *Streptococcus viridans* (gram positive bacteria); E.c. = *Escherichia coli(* gram negative bacteria) ; F.o. = *Fusarium oxysporium* (Fungus); A.a. = *Alternaria alternata* (Fungus); A.s. = *Alternaria solani*(Fungus); biochemical properties of the molecules are greater when metallation takes place. The results inferred that all the compounds were active against gram positive bacteria while less active against gram negative bacteria (*E. coli*). These mixed ligand di-n-butyltin(IV) complexes are more active as compared to the parent organic moieties which indicates that metallation increases the antimicrobial activity/46/. Although it is difficult to deduce an exact structure-activity relationship between the structure of these complexes and their microbial activities but the high activity of these complexes may be explained on the basis of the chelation theory /47/. Due to chelation of the metal ion, the polarity is reduced considerably on account of the partial sharing of its positive charge with the donor groups and the π electron delocalization over the whole chelate ring system. This, in turn increases the lipophilic character of the metal favouring its permeation through the lipid layer of the fungal membrane.

***Quantitative Antimicrobial Activity Results***

The MIC values have been recorded for **1**, **4**, 7, **10** and the data have been summarised in Table 6. The MIC values of **1**, **4** and **7** against different pathogenic fungi and bacteria suggest that the complexes are strongly antibacterial but poorly antifungal. While **10** is as good an antifungal agent as the commercially available antifungal agent Nystatin, the biological activity of LH,AH and their metal complexes is expected to be a function of steric, electronic and pharmokinetic factors/48/.

**Table 6**

Quantitative antimicrobial assay results [MIC (μg $cm^{-3}$)].

| Compound | S.a. | S.v. | F.o. |
|---|---|---|---|
| $Bu_2SnL^1A^1$ (**1**) | 473 | 625 | 518 |
| $Bu_2SnL^2A^1$ (**4**) | 331 | 165 | 16,500 |
| $Bu_2SnL^3A^1$ (7) | 312 | 156 | 18,400 |
| $Bu_2SnL^4A^1$ (**10**) | 781.0 | 391.0 | 12,500 |
| Kanamycin (antibacterial control) | 100 | >400 | |
| Nystatin (antifungal control) | | | 3135 |

MIC = minimum inhibitory concentration

### CONCLUSION

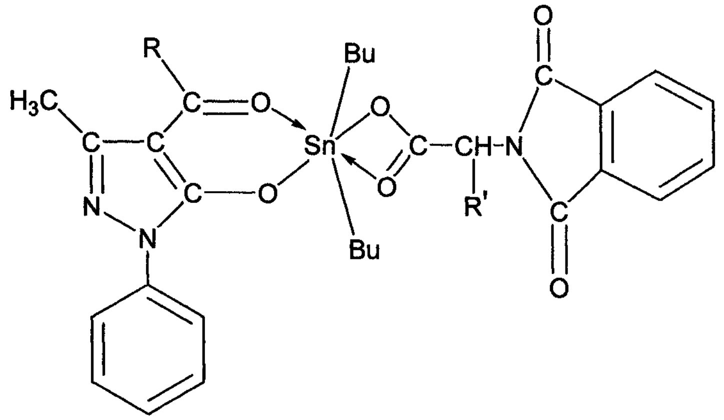

where R = $-4\text{-F-}C_6H_4\text{-}$, $-4\text{-Cl-}C_6H_4\text{-}$, $-4\text{-Br-}C_6H_4\text{-}$, $-CF_3$

R′ = -H, $-CH_3$, $-CH(CH_3)_2$

**Fig. 3**

These newly synthesised complexes of the type $Bu_2Sn[RCOC{:}C(O)N(C_6H_5)N{:}CCH_3][O_2CCHR'NC(O)C_6H_4C(O)]$ where R = $-4\text{-F-}C_6H_4\text{-}$ , - $4\text{-Cl-}C_6H_4\text{-}$, $-4\text{-Br-}C_6H_4\text{-}$, $-CF_3$ and R′ = -H, $-CH_3$, $-CH(CH_3)_2$ are found to be monomeric in nature. The heterocyclic β-diketones (LH) and N-phthaloyl amino acids (AH) have been suggested as bidentate O O donor chelating moieties. In these derivatives C-Sn-C lies in the range of 149.88° to 156.84° found for di-n-butyltin(IV) chelates in which the $^n$Bu substituents do not adopt cis or trans geometries about the central tin atom. It is best described as a skew trapezoidal bipyramidal geometry (**Fig. 3**).

### REFERENCES

1. M.S. Singh and K. Tawade, *Synth. React. Inorg. Met.-Org. Chem.*, **30**, 1015 (2000).
2. D. Agustin, G. Rima, H. Gornitzka and J. Barrsau, *Inorg. Chem.*, **39**, 5492 (2000).
3. H.L. Singh and A.K. Varshney, *Bull. Pol. Acad. Sci. Chem.*, **48**, 125 (2000).
4. D.-D. Yin, Y.-L. Jiang and Lu. Shan, *Chinese J. Chem.*, **19**, 1136 (2001).
5. A. Kriza and C. Parnau, *Acta Chim. Slovenica*, **48**, 445 (2001).
6. D.K. Dey, N. Bhartiya, R.K. Bansal and M.K. Das, *Main Group Met. Chem.*, **22**, 115 (1999).
7. M. Gielen, *Coord. Chem. Rev.*, **151**, 41 (1996).
8. L. Pellerito and L. Nagy, *Coord. Chem. Rev.*, **224**, 111 (2001).
9. S. Belwal, S.C. Joshi and R.V. Singh, *Main Group Met. Chem.*, **20**, 313 (1997).
10. J.C. Christine and A. Roy, *Inorg. Chim. Acta*, **107**, 57 (1985).
11. M. Gielen, E. Joosen and T. Mancilla, *Appl. Organomet. Chem.*, **16**, 48 (2002).
12. M. Gielen, E. Joosen and T. Mancilla, *J. Braz. Chem. Soc.*, **14**, 870 (2003).
13. M.S. Raizada and M.N. Srivastava, *Synth. React. Inorg. Met.-Org. Chem.*, **22**, 393 (1992).
14. A.M. Rouhi, *Chem. Eng. News*, **76**, 41 (1998).

15. R. Apodaka and W. Xiaq, *Org. Lett.*, **3**, 1745 (2001).
16. W.P. Neumann, *J. Organomet. Chem.*, **437**, 23 (1992).
17. R.J. Davey and A.M.M. Rebello, *J. Cryst. J. Cryst. Growth*, **1**, 187 (2001).
18. D. Morton, V. Russo, D. Stivanello and G. Tagliavini, *Organometallics*, **15**, 1645 (1996).
19. A. Joshi, S. Verma, A. Jain and S. Saxena, *Main Group Met. Chem.*, **27**, 123 (2004).
20. S. Verma, A. Joshi, A. Jain and S. Saxena, *J. Chem. Res.*, 768 (2004).
21. D.D. Perrin, W.L.F. Armarego and D.L. Perrin, *Purification of Laboratory Chemicals*, 3rd edn. Pergamon : London, 1988.
22. B.S. Jensen, *Acta Chem. Scand.*, **13**, 1347, 1668, 1890 (1959).
23. J.C. Sheehan, D.W. Chapman and R.W. Roth, *J. Am. Chem. Soc.*, **74**, 3822 (1952).
24. M.E. Hossain, M.N. Alam, M.A. Ali, M. Nazimuddin, F.E. Smith and R.E. Hayes, *Polyhedron*, **15**, 973 (1996).
25. A.M. Ali, M.M. Mackeen, I.I. Safinar, M. Hamid, N.H. Lajis, S.H. El-Sharkawy and M. Murakoshi, *J. Ethnopharmacology*, **53**, 165 (1996).
26. C.D. Hufford and A.M. Clark, in Atta-ur-Rahman, (Ed.), *Studies in Natural Products Chemistry*, Elsevier, Amsterdam, 1988, p. 421.
27. K. Nakamoto, *Infrared and Raman Spectra of Inorganic and Coordination Compounds*, 3rd edn. (John Wiley and Sons, New York), 1978.
28. R.C. Mehrotra and B.P. Bachlas, *J. Organomet. Chem.*, **22**, 121 (1970).
29. A.K. Saxena, S. Saxena and A.K. Rai, *Synth. React. Inorg. Met.-Org. Chem.*, **20**, 21 (1990).
30. A.K. Saxena, S. Saxena and A.K. Rai, *Indian J. Chem.*, **29A**, 255 (1990).
31. G.K. Sandhu, R. Hundal and E.R.T. Tiekink, *J. Organomet. Chem.*, **430**, 15 (1992).
32. C. Sreelatha and V.D. Gupta, *Synth. React. Inorg. Met.-Org. Chem.*, **1**, 29 (1988).
33. S.S. Sandhu, S.S. (Jr.) Sandhu, G.K. Sandhu, R.V. Parish and O. Parry, *Inorg. Chim. Acta*, **58**, 251 (1982).
34. M.K. Das, M. Nath and J.J. Zuckerman, *Inorg. Chim. Acta*, **71**, 49 (1983).
35. B.K. Agarwal, Y.P. Singh and A.K. Rai, *Indian J. Chem.*, **28A**, 912 (1989).
36. T.P. Lockhart and W.F. Manders, *Inorg. Chem.*, **25**, 892 (1986).
37. T.P. Lockhart and W.F. Manders, *J. Am. Chem. Soc.*, **109**, 7015 (1987).
38. B. Bovio, A. Cingolani, F. Marchetti and C. Pettinari, *J. Organomet. Chem.*, **484**, 123 (1994).
39. T.N. Mithell, *J. Organomet. Chem.*, **59**, 189 (1973).
40. J. Holocek and A. Lycka, *Inorg. Chim. Acta*, **118**, L15 (1986).
41. W.F. Jr. Howard, R.W. Crecely and W.H. Nelson, *Inorg. Chem.*, **24**, 2204 (1985).
42. D.L. Kepert, *Inorg. Chem.*, **12**, 1 (1977).
43. Z.-K. Yu, S.-H. Wang, Z.-Y. Yang and X.-M. Liu, *J. Organomet. Chem.*, **447**, 189 (1993).
44. B.A. Uzoukwu, *Indian J. Chem.*, **30A**, 372 (1991).
45. V.K. Jain, J. Mason, B.S. Saraswat and R.C. Mehrotra, *Polyhderon*, **4**, 2089 (1985).
46. H.L. Singh, B. Khungar, U.D. Tripathi and A.K. Varshney, *Main Group Met. Chem.*, **24**, 5 (2001).
47. B.G. Tweedy, *Phytopathology*, **55**, 910 (1964).
48. L. Mishra, *J. Indian Chem. Soc.*, **76**, 175 (1999) and references therein.
49. J. Holocek, M. Nadvornik, K. Handlir and A. Lycka, *J. Organomet. Chem.*, **315**, 29(1986).

